# Applications of Clinical Decision Support Systems in Diabetes Care: Scoping Review

**DOI:** 10.2196/51024

**Published:** 2023-12-08

**Authors:** Shan Huang, Yuzhen Liang, Jiarui Li, Xuejun Li

**Affiliations:** 1 Endocrinology Department Tongren Hospital Shanghai Jiao Tong University School of Medicine Shanghai China; 2 Department of Endocrinology The Second Affiliated Hospital Guangxi Medical University Nanning China; 3 Department of Endocrinology Cangzhou Central Hospital Cangzhou China; 4 Department of Endocrinology and Diabetes, The First Affiliated Hospital of Xiamen University School of Medicine, Xiamen University Xiamen China; 5 Xiamen Diabetes Institute Xiamen China; 6 Fujian Provincial Key Laboratory of Translational Medicine for Diabetes Xiamen China

**Keywords:** scoping review, clinical decision support system, CDSS, diabetes care, health information technology, clinical decision support, decision, decision support, diabetes, clinical application, decision-making, medical resources

## Abstract

**Background:**

Providing comprehensive and individualized diabetes care remains a significant challenge in the face of the increasing complexity of diabetes management and a lack of specialized endocrinologists to support diabetes care. Clinical decision support systems (CDSSs) are progressively being used to improve diabetes care, while many health care providers lack awareness and knowledge about CDSSs in diabetes care. A comprehensive analysis of the applications of CDSSs in diabetes care is still lacking.

**Objective:**

This review aimed to summarize the research landscape, clinical applications, and impact on both patients and physicians of CDSSs in diabetes care.

**Methods:**

We conducted a scoping review following the Arksey and O’Malley framework. A search was conducted in 7 electronic databases to identify the clinical applications of CDSSs in diabetes care up to June 30, 2022. Additional searches were conducted for conference abstracts from the period of 2021-2022. Two researchers independently performed the screening and data charting processes.

**Results:**

Of 11,569 retrieved studies, 85 (0.7%) were included for analysis. Research interest is growing in this field, with 45 (53%) of the 85 studies published in the past 5 years. Among the 58 (68%) out of 85 studies disclosing the underlying decision-making mechanism, most CDSSs (44/58, 76%) were knowledge based, while the number of non-knowledge-based systems has been increasing in recent years. Among the 81 (95%) out of 85 studies disclosing application scenarios, the majority of CDSSs were used for treatment recommendation (63/81, 78%). Among the 39 (46%) out of 85 studies disclosing physician user types, primary care physicians (20/39, 51%) were the most common, followed by endocrinologists (15/39, 39%) and nonendocrinology specialists (8/39, 21%). CDSSs significantly improved patients’ blood glucose, blood pressure, and lipid profiles in 71% (45/63), 67% (12/18), and 38% (8/21) of the studies, respectively, with no increase in the risk of hypoglycemia.

**Conclusions:**

CDSSs are both effective and safe in improving diabetes care, implying that they could be a potentially reliable assistant in diabetes care, especially for physicians with limited experience and patients with limited access to medical resources.

**International Registered Report Identifier (IRRID):**

RR2-10.37766/inplasy2022.9.0061

## Introduction

In 2021, the International Diabetes Federation (IDF) reported that around 537 million adults worldwide had diabetes [1], resulting in 6.7 million related deaths and US $966 billion ($1838.40 per capita) in total health expenditure [2]. Achieving target glucose levels for the treatment of diabetes can be challenging, as patients might lack knowledge about their condition and health care providers (HCPs) might face limitations, such as inadequate information, time, and support for making decisions [3,4]. Poor glycemic control can lead to an elevated propensity for complications associated with diabetes and cardiovascular disease (CVD) events, ultimately resulting in a reduction in life expectancy [5-10]. Combined with the rising prevalence of diabetes [1,2] and a scarcity of specialized endocrinologists [11], the use of clinical decision support systems (CDSSs) in diabetes care has become increasingly necessary to improve the health care of patients with diabetes.

CDSSs are defined as “technology-based systems that intend to improve health care delivery by enhancing medical decisions with targeted clinical knowledge, patient information, and other health information” [12]. According to their decision-making mechanisms, CDSSs are commonly classified into knowledge- or non-knowledge-based systems. The decision-making mechanism of knowledge-based CDSSs is based on explicit, predetermined knowledge rules or guidelines [13], whereas non-knowledge-based CDSSs use artificial intelligence (AI) or machine learning (ML) algorithms to transform large-scale health care data into meaningful information for users to make decisions [12,14]*.* Several reviews have been published, discussing the applications of CDSSs in the field of diabetes care. Some reviews in which only randomized controlled trials (RCTs) were included addressed precise questions, such as the effectiveness of CDSSs in diabetes care [15,16]. Some reviews focused on the use of CDSSs in specific patients or settings, such as inpatients with diabetes in the noncritical care setting [17,18], patients with type 1 diabetes [19-21], and patients with type 2 diabetes in primary care [22]. However, a comprehensive analysis of the application of CDSSs in diabetes care is still lacking.

Although CDSSs are a rapidly adopted and emerging technology in the field of diabetes care, some HCPs are still relatively unfamiliar with them in terms of applications in managing and treating diabetes [23]*.* CDSSs can promote diabetes care by facilitating patient self‐management [24] and improving the process of medication management [19]. Obtaining a comprehensive understanding of their current applications is critical, which could provide valuable insights to enable further development and optimal use of CDSSs in diabetes care. Therefore, we conducted a scoping review (ScR) with the aim of summarizing the landscape of the research status, clinical applications, and impact of CDSSs on both patients and physicians in diabetes care. ScRs synthesize information across a range of study types and designs and provide a broad overview of a topic [25,26]. Therefore, an ScR was more suitable for our study objective compared to systematic reviews, which focus on addressing more specific questions based on particular criteria of interest.

## Methods

### Study Design

The methodology of this ScR was based on the method described by Arksey and O’Malley [27], and the report followed the PRISMA-ScR (Preferred Reporting Items for Systematic Reviews and Meta-Analyses Extension for Scoping Reviews) guidelines. The protocol was developed and registered in the International Platform of Registered Systematic Review and Meta-analysis Protocols (INPLASY; #202290061).

### Determining the Research Question

This ScR aimed to answer 3 research questions (RQs):

- RQ1: What are the research characteristics of the applications of CDSSs in diabetes care?
- RQ2: What are the characteristics and clinical applications of CDSSs in diabetes care?
- RQ3: What is the impact of using CDSSs in diabetes care, and how can the impact be evaluated?

### Identifying Relevant Studies

This study aimed to include all relevant literature and conference abstracts in English or Chinese. To identify relevant studies, an extensive search was conducted across 7 electronic databases: PubMed, Embase, the Cochrane Library, the Web of Science, the China National Knowledge Infrastructure (CNKI), Wanfang, and VIP. All searches were performed from the date of database establishment up to June 30, 2022. In addition, searches of additional sources, such as Google, Baidu, and official conference websites, were conducted for academic conference abstracts from the period of 2021-2022. Details are listed in Multimedia Appendix 1.

### Study Selection

Studies were included if they reported the clinical application of CDSSs in diabetes care. CDSSs in this ScR referred to any technology-based systems (ie, mobile/tablet, web-based, or computer-based app) that can provide support for clinical decision-making and be applied across the whole spectrum of diabetes care, such as CDSSs used for treatment recommendation, complication risk assessment, and blood glucose monitoring.

The exclusion criteria were as follows:

- Studies published in languages other than Chinese or English
- Studies reporting CDSS technologies, algorithms, or theories and studies not directly pertaining to clinical decision support
- Studies not using clinical data (eg, genomic or protein data, or simulation data sets)
- Duplicate publications, research plans, reviews, commentaries, etc

Two researchers (authors XL and YL) independently evaluated the titles and abstracts of the identified studies based on the eligibility criteria. The full texts of potentially eligible studies were retrieved and then independently screened by the same 2 researchers (XL and YL). The 2 researchers also recorded the reasons for exclusion, and disagreements were resolved by a third senior researcher (author SH).

### Data Charting

Two researchers (authors SH and JL) independently collected data using a standardized data sheet. Disagreements were resolved by a third researcher (XL). We collected the following variables that were pertinent to the aims of this research: (1) study characteristics (eg, publication year, number of subjects, follow-up period), (2) CDSS characteristics (eg, decision-making mechanism, functions) and clinical applications, and (3) evaluation of CDSSs in diabetes care (eg, user experience, user adherence, effectiveness and safety).

### Collating, Summarizing, and Reporting Results

Continuous variables were summarized into categories to allow for a more meaningful summary. Categorical variables were summarized using frequency counts and percentages. The number of papers reporting the corresponding outcome was used as the denominator for each variable.

## Results

### Search and Selection

**Figure 1:**
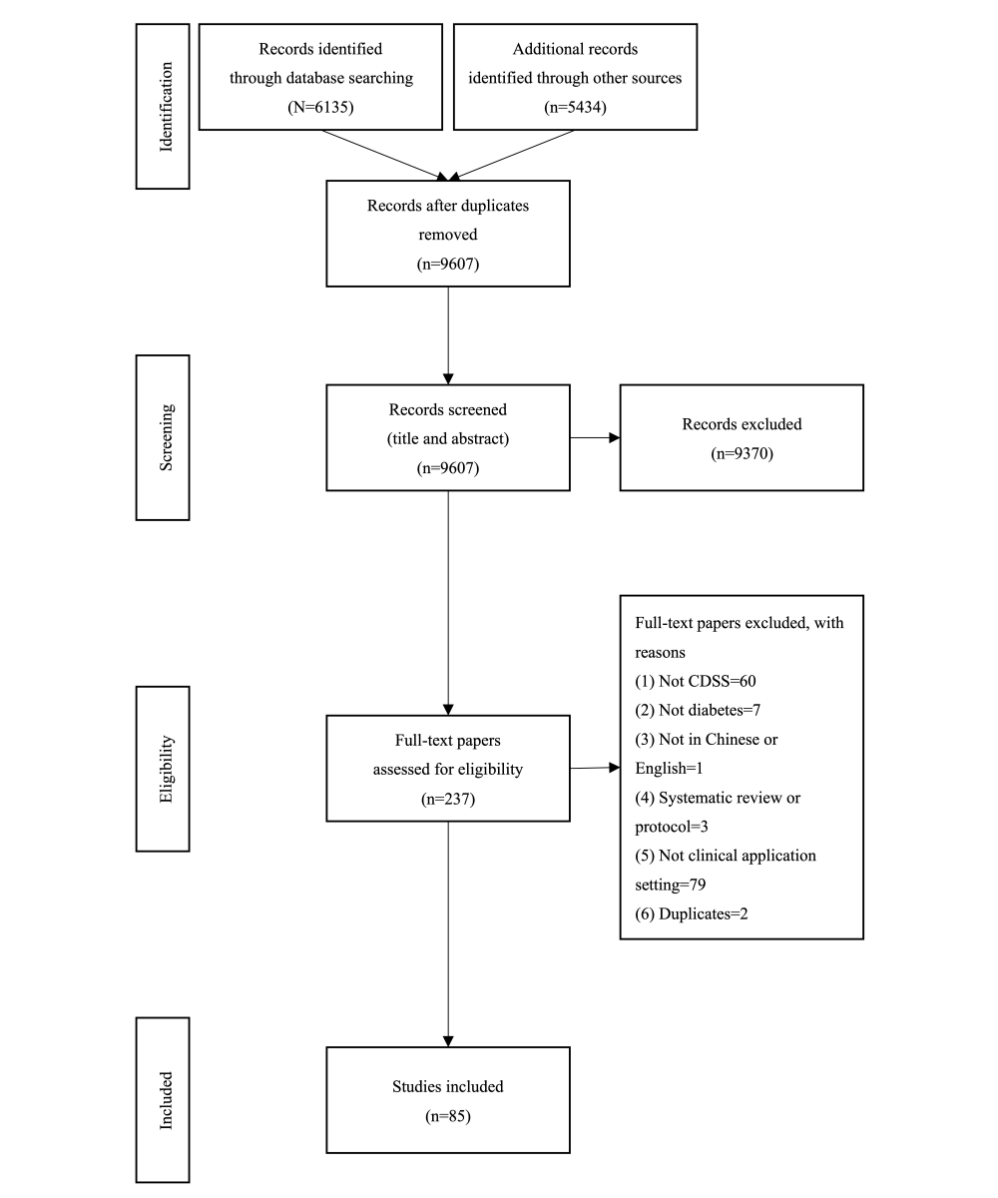
PRISMA-ScR flowchart demonstrating the study selection process. CDSS: clinical decision support system; PRISMA-ScR: Preferred Reporting Items for Systematic Reviews and Meta-Analyses.

A total of 11,569 studies were identified from included sources. After removing duplicated publications, 9607 (83%) studies were available for title and abstract screening. Finally, 237 (2.5%) studies were evaluated based on their full text, and 85 (35.9%) studies [23,28-111] (including 13/18, 15%, conference abstracts) were selected for analysis. The PRISMA-ScR flow diagram is shown in Figure 1 and the PRISMA-ScR checklist in Multimedia Appendix 2.

### Study Characteristics

In the 85 studies included in this ScR, a total of 159,475 subjects were enrolled. The number of publications was undergoing rapid growth during 2021-2022, and 45 (53%) of the studies were published in the past 5 years (2018-2022), as shown in Multimedia Appendix 3). Tables 1-7 summarize the characteristics of the included studies. The most common follow-up period was less than 12 months (<6 months: 20/75, 27%; 6-12 months: 38/75, 51%). The majority of studies were conducted in North American or European countries, with 34% (22/64) being conducted in the United States and 9% (6/64) in Austria and Germany. About half of the studies (34/65, 52%) were multicenter and 48% (31/65) were single-center studies. Most studies were RCTs (36/85, 42%), followed by single-arm trials (22/85, 26%), observational studies (18/85, 21%), and pragmatic clinical trials (PCTs; 5/85, 6%).

**Table 1:** Summary of study characteristics (N=85).

Characteristic and categories for each characteristic			Valid studies, n (%)^a,b^		References
**Publication years**					
	1986-1992	3 (4)		[28-30]	
	1993-1997	3 (4)		[31-33]	
	1998-2002	3 (4)		[34-36]	
	2003-2007	8 (9)		[37-44]	
	2008-2012	9 (11)		[45-53]	
	2013-2017	14 (16)		[54-67]	
	2018-2022	45 (53)		[23,68-111]	
**Number of subjects**					
	<100	32 (38)		[28-30, 32-34, 36, 37, 43, 57, 60, 63, 64, 67-69, 71, 73, 77, 80, 88, 92, 95, 97-99, 102, 103, 106, 108-110]	
	100-500	24 (28)		[23, 39, 40, 42, 46, 48, 49, 51, 52, 56, 59, 61, 62, 65, 75, 82-84, 87, 90, 94, 100, 104, 111]	
	501-999	13 (15)		[31, 38, 41, 45, 53-55, 72, 91, 93, 101, 105, 107]	
	≥1000	16 (19)		[35, 44, 47, 50, 58, 66, 70, 74, 76, 78, 79, 81, 85, 86, 89, 96]	

^a^Number of studies and percentages were presented. The percentages of valid, unspecified, and unapplicable studies were calculated within the total studies (N=85), while the percentages for each characteristic were calculated within the valid studies.

^b^Studies that reported the corresponding characteristics were considered valid studies.

**Table 2:** Summary of the baseline start year (N=85).

Characteristic and categories for each characteristic		Studies, n (%)^a^	References
**Valid studies (n=43, 51%)^b^**			
	1993-1997	1 (2)	[35]
	1998-2002	6 (14)	[38-42, 45]
	2003-2007	3 (7)	[44, 47, 50]
	2008-2012	8 (19)	[48, 49, 56, 58, 60, 70, 72]
	2013-2017	15 (35)	[57, 61, 63, 66, 73, 75, 78, 81, 84, 85, 87, 90, 93, 98, 108]
	2018-2022	10 (23)	[23, 79, 80, 83, 86, 96, 101, 103, 105, 107]
Unspecified studies		41 (48)	[28-34, 36, 37, 43, 46, 51-54, 59, 62, 64, 65, 67-69, 71, 74,76,77,82, 88, 89, 91, 92, 94, 95, 97, 99, 100, 102, 104, 106, 109, 111]
Unapplicable studies^c^		1 (1)	[110]

^a^Number of studies and percentages were presented. The percentages of valid, unspecified, and unapplicable studies were calculated within the total studies (N=85), while the percentages for each characteristic were calculated within the valid studies.

^b^Studies that reported the corresponding characteristics were considered valid studies.

^c^Computer simulation study.

**Table 3:** Summary of the follow-up period^a^ (N=85).

Characteristic and categories for each characteristic			Studies, n (%)^b^		References
**Valid studies (n=75, 88%)^c^**					
	<6 months	20 (27)		[28, 30, 32, 40, 43, 48, 52, 59, 63, 64, 69, 71, 77, 80, 83, 86, 97, 99, 104, 109]	
	6-11 months	38 (51)		[31, 33, 36-39, 41, 45-47, 49-51, 55-57, 61, 62, 65, 68, 74-76,78, 81, 84, 85, 87, 88, 92, 95, 96, 100-102, 107, 108, 111]	
	12-24 months	10 (13)		[23, 29, 34, 35, 42, 44, 53, 79, 93, 98]	
	>24 months	7 (9)		[54, 58, 60, 66, 70, 72, 90]	
Unspecified studies			6 (7)		[67, 73, 82, 89, 91, 94]
Unapplicable studies^d^			4 (5)		[103, 105, 106, 110]

^a^In 3 (4%) studies, the study design was a randomized controlled trial (RCT) cross-over; the follow-up period was defined as the overall study duration including the washout period for cross-over studies.

^b^Number of studies and percentages were presented. The percentages of valid, unspecified, and unapplicable studies were calculated within the total studies (N=85), while the percentages for each characteristic were calculated within the valid studies.

^c^Studies that reported the corresponding characteristics were considered valid studies.

^d^Computer simulation study and cross-sectional study.

**Table 4:** Summary of the country^a^ (N=85).

Characteristic and categories for each characteristic			Studies, n (%)^b^		References
**Valid studies (n=64, 75%)^c^**					
	United States	22 (34)		[23, 31, 33, 34, 41, 42, 50, 51, 55, 59, 66, 69, 70, 74, 79, 84-86, 98, 103, 104, 106]	
	Austria	6 (9)		[57, 60, 67, 73, 90, 94]	
	Germany	6 (9)		[30, 36, 43, 46, 84, 87]	
	China	5 (8)		[39, 63, 76, 101, 105]	
	South Korea	5 (8)		[40, 49, 61, 75, 103]	
	Spain	5 (8)		[53, 65, 88, 103, 107]	
	India	4 (6)		[58, 72, 78, 93]	
	United Kingdom	3 (5)		[32, 64, 88]	
	Canada	3 (5)		[45, 80, 108]	
	Israel	3 (5)		[84, 103, 104]	
	Belgium	2 (3)		[81, 103]	
	Netherlands	2 (3)		[44, 47]	
	Other^d^	16 (25)		[35, 44, 54, 56, 62, 72, 83, 84, 100, 103]	
Unspecified studies			21 (25)		[28, 29, 37, 38, 48, 52, 68, 71, 77, 82, 89, 91, 92, 95-97, 99, 102, 109-111]

^a^Of the valid studies, 5 (8%) were cross-national studies; thus, the sum of percentages may exceed 100%.

^b^Number of studies and percentages were presented. The percentages of valid, unspecified, and unapplicable studies were calculated within total studies (N=85), while the percentages for each characteristic were calculated within the valid studies.

^c^Studies that reported the corresponding characteristics were considered valid studies.

^d^Brazil, France, Ireland, Japan, Italy, Norway, Russia, Sri Lanka, Croatia, Finland, Serbia, Greece, the Czech Republic, the United Arab Emirates, Pakistan, and Slovenia (1 study for each country).

**Table 5:** Summary of the number of centers (N=85).

Characteristic and categories for each characteristic			Studies, n (%)^a^		References
**Valid studies (n=65, 77%)^b^**					
	Multicenter	34 (52)		[23, 31, 35, 36, 43, 44, 47, 51-53, 58, 59, 62, 63, 65, 72, 74-77, 79-81, 83, 84, 88, 89, 98, 100, 103, 104, 106-108]	
	Single center	31 (48)		[30, 32-34, 38-42, 45,49, 50, 54-57, 60, 61, 64, 66-68, 70, 73, 78, 85, 87, 90, 94, 96, 105]	
Unspecified studies			18 (21)		[28, 29, 37, 48, 69, 71, 82, 91-93, 95, 97, 99, 101, 102, 109-111]
Unapplicable studies^c^			2 (2)		[46, 86]

^a^Number of studies and percentages were presented. The percentages of valid, unspecified, and unapplicable studies were calculated within the total studies (N=85), while the percentages for each characteristic were calculated within the valid studies.

^b^Studies that reported the corresponding characteristics were considered valid studies.

^c^Database studies.

**Table 6:** Summary of the study design (N=85).

Characteristic and categories for each characteristic		Valid studies, n (%)^a,b^	References
**Observational studies (n=18, 21%)**			
	Prospective cohort study	5 (28)	[23, 29, 72, 97, 107]
	Retrospective cohort study	9 (50)	[46, 54, 55, 70, 86, 87, 91, 101, 102]
	Ambispective cohort study	1 (6)	[65]
	Cross-sectional study	3 (17)	[103, 105, 106]
**RCTs^c^ (n=36, 42%)**			
	Parallel design	23 (64)	[28, 30-33, 35, 36, 38-40, 42, 43, 49, 56, 61, 63, 74, 75, 80, 83, 84, 93, 98]
	Cross-over design	3 (8)	[69, 71, 88]
	Cluster RCT	10 (28)	[41, 47, 50-53, 66, 81, 85, 89]
PCT^d^		5 (6)	[44, 45, 59, 78, 108]
Single-arm trial		22 (26)	[34, 37, 48, 57, 58, 62, 64, 67, 68, 73, 76, 77, 79, 92, 94-96, 99, 100, 104, 109, 111]
Other^e^		4 (5)	[60, 82, 90, 110]

^a^Number of studies and percentages were presented. The percentages of valid, unspecified, and unapplicable studies were calculated within the total studies (N=85), while the percentages for each characteristic were calculated within the valid studies.

^b^Studies that reported the corresponding characteristics were considered valid studies.

^c^RCT: randomized controlled trial.

^d^PCT: pragmatic clinical trial.

^e^Post hoc analysis and computer simulation.

**Table 7:** Summary of the data source (N=85).

Characteristic and categories for each characteristic			Valid studies, n (%)^a,b^		References
Primary data			68 (80)		[23, 28-45, 47-53, 56-59, 61-69, 71-81, 83-85, 88, 89, 93, 94, 96-98, 101, 104-109, 111]
**Secondary data (n=22, 26%)**					
	EHR^c^	10 (36)		[48, 54, 55, 65, 90-92, 100, 102, 103]	
	Devices	6 (27)		[87, 95, 99, 101, 103, 108]	
	Clinical trials	4 (18)		[60, 82, 90, 110]	
	Surveys	3 (14)		[46, 48, 62]	
	Patient-reported outcomes	2 (9)		[45, 70]	
	Registry	1 (5)		[46]	

^a^Number of studies and percentages were presented. The percentages of valid, unspecified, and unapplicable studies were calculated within the total studies (N=85), while the percentages for each characteristic were calculated within the valid studies.

^b^Studies that reported the corresponding characteristics were considered valid studies.

^c^EHR: electronic health record.

### Characteristics and Clinical Applications of CDSSs in Diabetes Care

The characteristics of CDSSs are summarized in Table 8. Most CDSSs included in this ScR were knowledge based (44/58, 76%), although non-knowledge-based CDSSs have been increasing in recent years. In 59% (48/82) of the studies, physicians were the users of CDSSs. In 51% (42/82) of the studies, patients were the users of CDSSs. Both physicians and patients were the users of CDSSs in 10% (8/82) studies. In 30% (25/82) of the studies, nurses, medical assistants, and pharmacists supported physicians in using CDSSs. The types of outputs provided by CDSSs were vast, and we classified them based on the 6 categories of intervention types reported in the *Clinical Decision Support Implementers’ Workbook* [112]. Most CDSSs facilitated users by providing proactive order suggestions and order sets (65/83, 78%) and supporting guidelines, complex protocols, algorithms, and clinical pathways (25/83, 30%).

**Table 8:** Summary of CDSS^a^ characteristics.

Characteristic and categories for each characteristic		Studies, n (%)^b^	References
**Decision-making mechanism^c^: valid studies^d^ (n=58, 68%)**			
	Knowledge based	44 (76)	[23, 28, 30-33, 35, 36, 38, 40, 42-47, 49, 50, 53, 54, 58, 59, 61, 65, 66, 70, 72, 74, 78, 79, 81, 83, 96, 97, 100, 106, 108, 109]
	Non-knowledge based	14 (24)	[37, 57, 64, 69, 71, 73, 80, 84, 85, 88, 103-105, 107]
Decision-making mechanism: unspecified studies		27 (32)	[29, 34, 39, 41, 48, 51, 52, 55, 60, 62, 63, 68, 75, 77, 82, 86, 89, 91-93, 95, 98, 99, 101, 102, 110, 111]
**Setting: valid studies (n=73, 86%)**			
	Primary care	29 (40)	[23, 28, 29, 33, 35, 42, 44, 45, 47, 50, 52-55, 58, 59, 62, 63, 65, 66, 74, 78, 79, 81, 83, 85, 89, 104, 106]
	Specialized hospital	23 (32)	[31, 34, 38-40, 43, 56, 57, 60, 67, 73, 76, 77, 82, 84, 90, 94, 96, 97, 102, 103, 105, 107]
	Diabetes center	6 (8)	[30, 32, 68, 69, 72, 87]
	Household	15 (21)	[37, 49, 51, 61, 64, 75, 88, 92, 93, 95, 98, 99, 101, 108, 109]
Setting: unspecified studies		12 (14)	[36, 41, 46, 48, 70, 71, 80, 86, 91, 100, 110, 111]
**Target patient (type of diabetes): valid studies (n=85, 100%)**			
	Type 2 diabetes	42 (49)	[23, 38-41, 44, 45, 47, 49-54, 57, 59-63, 65-67, 72, 73, 75, 76, 78, 79, 81-83, 87, 90-94, 97, 102, 105, 107, 111]
	Type 1 diabetes	20 (24)	[28, 29, 34, 36, 37, 64, 69, 71, 80, 84, 86, 88, 95, 98, 99, 101, 103, 104,109, 110]
	Gestational diabetes	1 (1)	[100]
	Multiple types^e^	17 (20)	[30-33, 35, 42, 43, 46, 48, 55, 58, 68, 70, 74, 85, 96, 108]
	Other^f^	5 (6)	[56, 77, 89, 106]
**Target patient (age group [years]): valid studies (n=71, 84%)**			
	<20	5 (7)	[66, 80, 84, 95, 103]
	[20,40)	9 (13)	[30, 32, 34, 36, 37, 69, 86, 101, 110]
	[40,50)	7 (10)	[28, 29, 43, 56, 64, 71, 88]
	[50,60)	18 (25)	[40, 48, 51, 54, 55, 59, 65, 68, 70, 75, 78, 83, 85, 89, 91, 93, 98, 108]
	≥60	32 (45)	[35, 38, 39, 41, 42, 44-47, 49, 53, 57, 60-63, 67, 73, 74, 77, 79, 81, 82, 87, 90, 92, 94, 96, 102, 105, 107, 111]
Target patient (age group): unspecified studies		14 (16)	[23, 31, 33, 50, 52, 58, 72, 76, 97, 99, 100, 104, 106, 109]
**User type: valid studies (n=82, 96%)**			
	Patient	42 (51)	[28-30, 36, 37, 39-42, 45, 49, 51, 54, 56, 59, 61, 63-65, 68-71, 75, 84, 86-89, 91-93, 95, 98, 99, 101, 104, 106, 108-111]
	HCP^g^	48 (59)	[23, 32-35, 38, 42-48, 50, 52-55, 57, 58, 60, 62, 66, 67, 69, 70, 72-74, 76-81, 83-85, 89, 90, 94, 96, 97, 103, 105-107]
	Physician only	23 (28)	[31, 35, 43, 45, 46, 53-55, 66, 67, 69, 70, 76, 80, 81, 84, 85, 89, 96, 97, 103, 105, 107]
	Physician assisted by nurses, medical assistants, and pharmacists	25 (30)	[23, 32-34, 38, 42, 44, 47, 48, 50, 52, 57, 58, 60, 62, 72-74, 77-79, 83, 90, 94, 106]
User type: unspecified studies		3 (4)	[82, 100, 102]
**Function^h^: valid studies (n=83, 98%)**			
	Forms and templates	9 (11)	[31, 33, 41, 44, 52, 62, 66, 88, 108]
	Relevant data presentation	24 (29)	[31, 34, 40, 41, 49, 51, 52, 54, 61-63, 66, 71, 76, 84, 85, 87, 88, 96, 98, 101, 105-107]
	Proactive order suggestions and order sets	65 (78)	[23, 28-30, 32, 33, 35-50, 53, 54, 56-58, 60-62, 64, 65, 67-69, 72-84, 86, 88-90, 93-95, 97-99, 102-104, 106, 108-110]
	Support for guidelines, complex protocols, algorithms, clinical pathways	25 (30)	[31, 33, 35, 38, 40, 43-46, 50, 51, 53, 54, 61, 66, 72, 74, 76, 78, 81, 83, 87, 88, 96, 109]
	Reactive alerts	15 (18)	[31, 33, 38, 45, 49, 54, 61, 62, 70, 78, 81, 84, 93, 96, 108]
	Reference information and guidance	14 (17)	[33, 35, 42, 51, 54, 59, 61, 63, 81, 83, 91, 93, 101, 111]
Function: unspecified studies		2 (2)	[55, 100]

^a^CDSS: clinical decision support system.

^b^Number of studies and percentages were presented. The percentages of valid, unspecified, and unapplicable studies were calculated within the total studies (N=85), while the percentages for each characteristic were calculated within the valid studies.

^c^Knowledge-based CDSSs used explicit, predetermined knowledge rules or guidelines [13], whereas non-knowledge-based CDSSs used artificial intelligence (AI) or machine learning (ML) algorithms.

^d^Studies that reported the corresponding characteristics were considered valid studies.

^e^Patients had both type 1 and type 2 diabetes.

^f^Healthy adults with a family history of type 2 diabetes, critically ill patients with hyperglycemia, or adults with prediabetes.

^g^HCP: health care provider.

^h^We classified the studies on CDSS use in diabetes care based on the 6 categories of intervention types reported by Osheroff et al [112]. Some studies combined several CDSS intervention types and therefore are represented in multiple categories.

Users could leverage CDSSs in the clinical management of diabetes in various ways (Table 9). The most common application scenario of CDSSs was to provide treatment recommendations (63/81, 78%), not only for physicians (36/47, 77%), but also for patients (29/42, 69%). Of the 36 (77%) studies, CDSSs were commonly used by physicians for drug recommendations (n=22, 61%) and insulin dose adjustment (n=14, 39%). Of the 29 (69%) studies, CDSSs were used by patients not only for insulin dose adjustment (n=16, 55%) and drug recommendations (n=6, 21%) but also for suggestions for diet and exercise (n=11, 38%). Other application scenarios included medical education (13/81, 16%), complication risk assessment (12/81, 15%), blood glucose monitoring (12/81, 15%), diabetes screening (4/81, 5%), and appointments for examinations (3/81, 4%). When categorizing physician users according to their medical disciplines, primary care physicians (20/39, 51%) were the most common, followed by endocrinologists (15/39, 38%) and nonendocrinologists (8/39, 21%). For primary care physicians as users in 20 (51%) studies, CDSSs were mainly used for treatment recommendations (n=16, 80%) and no application scenario for blood glucose monitoring was found. For endocrinologists as users in 15 (38%) studies, CDSSs were mainly used for treatment recommendations (n=13, 87%) and no application scenarios for medical education, appointments for examinations, and diabetes screening were found. For nonendocrinologists as users in 8 (21%) studies, CDSSs were only used for treatment recommendations (n=4, 50%), complication risk assessment (n=3, 38%), and diabetes screening (n=1, 13%).

**Table 9:** Clinical applications of CDSSs^a^ in diabetes care by user.

Clinical application			All users^b^ (n=81)		Physicians as users									Patients as users
					All physicians (n=47)		Medical discipline^c^: primary care (n=20)		Medical discipline: specialist endocrinology (n=15)		Medical discipline: specialist nonendocrinology^d^ (n=8)		All patients (n=42)	
**Treatment recommendations**			63 (78)		36 (77)		16 (80)		13 (87)		4 (50)		29 (69)	
	Insulin dose adjustment	30 (48)		14 (39)		1 (6)		9 (69)		3 (75)		16 (55)		
	Drug recommendations^e^	27 (43)		22 (61)		15 (94)		4 (31)		1 (25)		6 (21)		
	Suggestions for diet and exercise	11 (17)		1 (3)		1 (6)		0		0		11 (38)		
Complication risk assessment^f^			12 (15)		9 (19)		4 (20)		2 (13)		3 (38)		4 (10)	
Medical education			13 (16)		4 (9)		3 (15)		0		0		10 (24)	
Appointments/alerts of examinations			3 (4)		3 (6)		1 (5)		0		0		2 (5)	
Diabetes screening in high-risk population			4 (5)		4 (9)		3 (15)		0		1 (13)		1 (2)	
Blood glucose monitoring			12 (15)		1 (2)		0		1 (7)		0		11 (26)	

^a^CDSS: clinical decision support system.

^b^Of the 85 studies included, 3 (4%) with missing information on user type and 1 (1%) with missing information on specific clinical application were excluded from the analysis. Numbers of studies and percentages are presented. Some CDSSs were used in multiple clinical applications. The percentages of subcategories of treatment recommendations were calculated within treatment recommendations. Both physicians and patients were users of CDSSs in 10% (8/81) studies.

^c^Of 47 studies, 39 (83%) reported the medical discipline of physicians and 8 (17%) reported missing relevant information about medical disciplines; Physicians were from multiple disciplines in 4 (10%) of 39 studies.

^d^Ophthalmology, neurology, cardiology, surgery, the emergency department, the intensive care unit, and pediatrics.

^e^Recommendations for antidiabetic drugs, antihypertensive drugs, and lipid-lowering drugs.

^f^Complications include cardiovascular disease (CVD), diabetes retinopathy, diabetes foot, renal failure, hyperglycemia, and hypoglycemia.

### Evaluation of CDSSs in Diabetes Care

CDSSs in diabetes care have been evaluated using various dimensions, including the effectiveness, safety, consistency, and diagnostic accuracy of CDSSs; user behavior, user adherence, and user experience; and cost-effectiveness. Studies that evaluated the effectiveness and safety of CDSSs regarding biomarkers were the most prevalent, and the results of the effectiveness of CDSSs for biomarkers are summarized in Table 10. Regarding the safety of using CDSSs, the risk of hypoglycemia significantly decreased in 34% (12/35) studies [36,49,60,61,67,69,73,82,86,87,98,110].

**Table 10:** Summary of the effectiveness (64/85, 75%) of CDSSs^a^ for biomarkers.

Outcomes		Studies that showed CDSSs significantly improved outcomes, n/N (%)^b^	References
**Blood glucose**		45/63 (71)	[23, 28-30, 32, 34, 36, 39, 42-46, 48-51, 53, 54, 57, 58, 61, 62, 65, 68, 69, 73-75, 78, 79, 82, 84, 86, 87, 90-94, 98, 101, 102, 104, 110]
	HbA1c^c^	30/43 (70)	[23, 28, 29, 34, 36, 39, 42-46, 49-51, 53, 54, 61, 62, 65, 68, 74, 75, 78, 79, 84, 86, 87, 91, 92, 98]
	FBG^d^	4/9 (44)	[39, 58, 93, 101]
	MBG^e^	8/14 (57)	[30, 32, 36, 43, 46, 82, 90, 102]
	TIR^f^	12/18 (67)	[48, 57, 73, 82, 87, 90, 94, 98, 101, 102, 104, 110]
	GV^g^	5/7 (71)	[36, 46, 48, 69, 87]
**Blood pressure**		12/18 (67)	[35, 42, 44, 45, 47, 50, 58, 61, 63, 65, 72, 78]
	SBP^h^	11/18 (61)	[42, 44, 45, 47, 50, 58, 61, 63, 65, 72, 78]
	DBP^i^	10/15 (67)	[35, 42, 44, 45, 47, 50, 58, 63, 65, 72]
**Blood lipid**		8/21 (38)	[39, 42, 44, 47, 61, 65, 72, 74]
	LDL^j^ cholesterol	6/15 (40)	[39, 44, 47, 65, 72, 74]
	HDL^k^ cholesterol	1/7 (14)	[39]
	TC^l^	4/11 (36)	[39, 44, 47, 61]
	TG^m^	2/5 (40)	[39, 42]

^a^CDSS: clinical decision support system.

^b^The results were represented as the ratio of the number of studies with a significant improvement in outcomes to the number of studies with related indicators.

^c^HbA1c: glycated hemoglobin.

^d^FBG: fasting blood glucose.

^e^MBG: mean blood glucose.

^f^TIR: time in range.

^g^GV: glucose variability.

^h^SBP: systolic blood pressure.

^i^DBP: diastolic blood pressure.

^j^LDL: low-density lipoprotein.

^k^HDL: high-density lipoprotein.

^l^TC: total cholesterol.

^m^TG: triglyceride.

Of the 85 studies included, 64 (75%) assessed the effectiveness of CDSSs in improving patients’ blood glucose (n=63, 98%), blood pressure (n=18, 28%), and blood lipid levels (n=21, 33%). Significant improvements in biomarkers were based on the reported results of the included studies. A significant improvement in any 1 biomarker was considered significant. CDSSs significantly improved patients’ blood glucose (45/63, 71%), blood pressure (12/18, 67%), and blood lipid (8/21, 38%) levels. Specifically, CDSSs significantly improved glycated hemoglobin (HbA1c; 30/43, 70%), glucose variability (GV; 5/7, 71%), diastolic blood pressure (DBP; 10/15, 67%), time in range (TIR; 12/18, 67%), systolic blood pressure (SBP; 11/18, 61%), and mean blood glucose (MBG; 8/14, 57%).

In addition, 35 (41%) studies evaluated the safety of CDSS use in diabetes care, indicating that CDSSs would not increase the risk of hypoglycemia. Meanwhile, CDSSs significantly decreased the risk of hypoglycemia in 34% (12/35) of the studies.

Furthermore, 3 (4%) studies analyzed the consistency of insulin dose adjustments determined between CDSS algorithms and physicians, suggesting that the recommendations made by CDSSs are like those made by physicians and have an acceptance rate of more than 90% among HCPs. Additionally, 2 (2%) studies demonstrated the diagnostic accuracy of CDSSs in predicting the risk of diabetic retinopathy.

Of the 85 studies included, 16 (19%) evaluated users’ adherence and the results are summarized in Multimedia Appendix 4. In 7 (44%) studies, users’ adherence to insulin dose suggestions was over 90%. In 7 (44%) studies, CDSSs improved users’ adherence to follow-up and examination appointments, diabetes care guidelines, and drug usage. In the remaining 2 (13%) studies, users’ adherence to suggestions for lifestyle changes ranged from 50% to 80%.

In addition, 25 (29%) studies evaluated the user experience of CDSSs. All studies reported positive comments, with 9 (36%) also reporting negative comments. Users provided favorable comments for CDSSs, such as “It was easy to use” [81] and “It offered useful information” [62]. Meanwhile, challenges and limitations associated with using CDSSs were exposed by negative comments, for example, “Software glitches influenced physicians’ acceptance of CDSSs” [23], “A lack of integration with the electronic health record (EHR) system would result in a more complicated workflow” [23], “Some recommendations provided by CDSSs did not consider comorbidities or patient adherence” [23], and “CDSSs were not up to date on the most recent guidelines” [72].

## Discussion

### Principal Findings

To the best of our knowledge, this is the first ScR to provide a comprehensive analysis of the use of CDSSs in diabetes care. Our findings suggest a significant increase in the number of studies and relevant study participants in recent years, reflecting a growing interest in using CDSSs in diabetes care. Most CDSSs are knowledge based, while the number of non-knowledge-based CDSSs has been increasing in recent years. CDSSs can be used by diverse users (even nonendocrinologists, nurses, medical assistants, and pharmacists) in various application scenarios, including treatment recommendations, medical education, complication risk assessment, blood glucose monitoring, appointments for examinations, and diabetes screening. The included studies demonstrated that CDSSs are effective and safe for diabetes care.

### CDSSs Could Be Effective and Safe in Improving Diabetes Care

Studies assessing the effectiveness of CDSSs primarily used biomarkers (eg, HbA1c, TIR, or low-density lipoprotein [LDL]) as endpoints. The most common follow-up period in the included studies was less than 12 months. Our review found that CDSSs significantly improve blood glucose, blood pressure, and lipid profile (71%, 67%, and 38% of the studies, respectively) and that the risk of hypoglycemia does not increase correspondingly. This aligns with the results of previous reviews [15,16,18,113-115]. In recent years, there has been an increasing focus on long-term outcomes in diabetes care [116-122]. The long-term outcomes of implementing CDSSs are still unknown; thus, further research with long-term outcomes is needed.

An evident disparity was observed between the care recommended by clinical guidelines and the actual care provided to patients, ultimately leading to suboptimal glycemic control outcomes [123]. CDSSs might play a vital role in improving the quality of diabetes care in the following ways:

- CDSSs were most commonly used to provide recommendations for insulin dose adjustment (30/81, 37%). For insulin users, it is critical to adjust the insulin dose properly and frequently according to patients’ blood glucose levels, physical activity, and dietary patterns, which requires patients to undergo frequent clinical visits and HCPs with clinical experience and expertise [103]. HCPs with limited clinical experience might find insulin dose adjustment to be a challenge. CDSSs leverage data (eg, glucose level, insulin delivery rate, and food intake) from patient devices to automatically generate precise insulin dosing recommendations. The recommendations provided by CDSSs closely resemble those provided by experienced physicians, and a high rate of agreement with these recommendations is observed among HCPs [80,103,104].
- The secondary application of CDSSs is to provide drug recommendations (27/81, 33%). Managing diabetes has become increasingly complex with the expansion of treatment options and the growing emphasis on personalized care strategies outlined in the guidelines [124]. This presents a challenge for HCPs, particularly primary care physicians, who must balance managing multiple chronic conditions within limited time constraints [125]. By integrating the latest clinical guidelines with patients’ clinical characteristics, CDSSs provide HCPs with advice on drug selection, improving their decision-making efficiency in developing individualized treatment plans.
- CDSSs have been shown to improve users’ adherence (eg, adherence to medication suggestions, care guidelines, and follow-up appointments), which might improve clinical inertia. Clinical inertia, defined as “the failure to initiate or intensify therapy in a timely manner according to evidence-based clinical guidelines in individuals who are likely to benefit from such intensification” [126], is common in diabetes care and is caused by multifaceted factors [126,127]. CDSSs for patients with diabetes provide blood glucose monitoring and medical education, which could strengthen patients’ awareness of their chronic conditions and increase patients’ willingness for treatment modification. CDSSs for HCPs offer valuable support via treatment recommendations and physician training, thereby enhancing the ability of HCPs to make optimal decisions based on the unique needs of each patient, facilitating them in promptly and effectively modifying treatment regimens.

### CDSSs Might Be More Required in Some Specific Contexts

CDSSs might be most useful for HCPs with limited formal education and practical experience in diabetes care or for patients with limited access to medical resources [84,128]. As the incidence of diabetes continues to rise and the number of qualified endocrinologists remains inadequate [11], primary care physicians might find themselves increasingly responsible for managing patients with diabetes [84,129]. Primary care physicians face challenges in diabetes management as they usually deal with multiple health issues and have little experience with the standard of care for diabetes [130]. Our review revealed that primary care physicians are the main users of CDSSs in diabetes care, especially in the scenario of drug recommendations, likely due to their lack of knowledge of the latest guidelines compared to specialized endocrinologists. Regarding glucose management, nonphysician HCPs (eg, nurses, medical assistants, and pharmacists) and nonendocrinologists face comparable situations to those of primary care physicians, suggesting that CDSSs have great potential for application in these situations. Our review found that some nurses, medical assistants, pharmacists, and nonendocrinologists have initiated the use of CDSSs in diabetes care.

Additionally, effective diabetes care is a multifaceted process that relies not only on the expertise of HCPs but also on the active participation of patients in managing their diet, exercise routine, medication management, and other important health factors [131]. Benefiting from the development of mobile internet technology, our review found an increasing trend of CDSSs being developed for patient-oriented care. These CDSSs could facilitate patient self-management in diverse application scenarios, such as providing recommendations for insulin dose adjustment, providing suggestions for diet and exercise, providing medical education, and monitoring blood glucose. This might be especially appealing to patients who live in rural areas or have limited access to in-person physician visits [84].

### Challenges and Prospects of CDSSs in Diabetes Care

Sirajuddin’ et al [132] stated that for modern CDSSs to be effective, they should follow the Five Rights model. This model emphasizes that delivering “the right information to the right person, in the right format, through the right channel, and at the right time” is crucial for achieving lasting improvements. This ScR found that not all CDSSs could fit the model.

One of the challenges identified in this study was the suboptimal format and channel, such as the lack of integration with hospital electronic systems and the unfavorable design of the CDSS’s human-computer interaction [23]. Integration of CDSSs into hospital systems to reduce physicians’ workload [133], expediting software iteration, and developing CDSSs in collaboration with physicians [134] could help resolve these challenges.

Some CDSSs in the studies included reported challenges in providing the “right information.” User acceptability of CDSS recommendations has decreased due to incomplete data collection [23] and delayed updates to the CDSS knowledge base [72]. Smart wearable devices could be leveraged to improve the efficiency and accuracy of data collection [135] and assist in making specific recommendations as opposed to a variety of suggestions. It is challenging to timely manage and maintain the rules (created based on expert knowledge, guidelines, etc) of CDSSs. Outdated rules could lead to inaccurate suggestions for treatments or preventive services. The extensive range of available data sets has led to the application of new methods (eg, association rule mining and machine learning algorithms) to explore novel modes of knowledge, which might reduce the cost of updating and maintaining the knowledge base [128].

In addition, our review found that although knowledge-based CDSSs remain the most commonly used type, the rise of AI and big data has led to an increase in non-knowledge-based CDSSs, which are primarily used to provide treatment recommendations for insulin dose adjustment and predict the patient’s risk of complications. It is possible that we are currently undergoing a transformation from the rule-based approach to new methods, such as machine learning combined with voluminous clinical databases, offering more precise and personalized approaches to health care [128,136].

Furthermore, CDSSs might be effective and safe in improving diabetes care, but the cost of design, local implementation, ongoing maintenance, and user support for CDSSs could be high [137,138], which might be a significant barrier to fully implementing CDSSs. In the view of service payers (eg, health care facilities, insurers, and policy makers) promoting the use of CDSSs, it is important to find evidence about whether CDSSs are cost-effective in improving diabetes care. However, few studies have reported the cost and economic benefits of CDSS implementation.

### Implications for Future Research

As discussed before, there are several research gaps. Future research should:

- Consider long-term follow-up to expand the range of outcomes, such as major adverse cardiovascular events (MACE), heart failure, and chronic kidney disease
- Investigate the use of CDSSs by nonphysician HCPs (eg, nurses, medical assistants, and pharmacists) and health care physicians not specialized in diabetes care
- Explore the implementation of CDSSs in diabetes care in cases of limited resources
- Evaluate the cost-effectiveness of CDSSs in diabetes care

### Limitations

This ScR has several limitations. First, publication bias could exist in the studies included as negative results may not always be published. Second, this ScR could be subject to information bias due to certain data being collected based on subjective judgment. However, senior researchers and experts participated in data validation and verification to minimize potential bias. For instance, it is difficult to distinguish between knowledge-based and non-knowledge-based CDSSs. To address this issue, we enlisted the assistance of industry professionals to identify the decision-making mechanisms of CDSSs, but misclassification might still exist. Lastly, the great heterogeneity in CDSSs’ design, purpose, and targets for evaluation prevented us from conducting a quality assessment and a meta-analysis, which according to ScR guidelines is usually not required.

### Conclusion

This ScR found that CDSSs are being increasingly used in diabetes care and have been widely implemented by diverse users across various scenarios. They have been shown to be effective and safe in improving diabetes care, implying that CDSSs can be a reliable assistant for physicians and might be particularly helpful for physicians with limited experience and patients with limited access to medical resources. CDSSs also face some challenges and necessitate ongoing optimization iterations. Future studies should focus on further improving CDSS performance, evaluating their long-term effects and cost-effectiveness, and promoting their usage among HCPs and patients beyond endocrinology.

## Appendix

Multimedia Appendix 1 Search strategy.

Multimedia Appendix 2 PRISMA-ScR checklist.

Multimedia Appendix 3 Number of publications and subjects over time (N=85).

Multimedia Appendix 4 Summary of users' adherence.

## Abbreviations

AI: artificial intelligence
CDSS: clinical decision support system
CVD: cardiovascular disease
DBP: diastolic blood pressure
EHR: electronic health record
GV: glucose variability
HbA1c: glycated hemoglobin
HCP: health care provider
LDL: low-density lipoprotein
MBG: mean blood glucose
ML: machine learning
PCT: pragmatic clinical trial
PRISMA-ScR: Preferred Reporting Items for Systematic Reviews and Meta-Analyses Extension for Scoping Reviews
RCT: randomized controlled trial
RQ: research question
SBP: systolic blood pressure
ScR: scoping review
TIR: time in range
