# Supplementary material for: Applications of Clinical Decision Support Systems in Diabetes Care: Scoping Review
Source: J Med Internet Res. 2023 Dec 8;25:e51024. doi: 10.2196/51024 (PMC10746969; doi:10.2196/51024)
Supplement: Multimedia Appendix 1 [file jmir_v25i1e51024_app1.doc]

**Appendix 1. Search strategy.**

| **Database** | **Search Strategy** |
| --- | --- |
| PubMed | #1 "clinical decision support system*" OR "cdss*" OR "decision support system*" OR "dss" OR "computer-assisted".ti.ab  #2 "diabetes" OR "DM".ti.ab  #3 #1 AND #2 |
| Embase | #1 'clinical decision support system*' OR 'cdss*' OR 'decision support system*' OR 'dss' OR 'computer-assisted':ti,ab,kw  #2 'diabetes' OR 'dm':ti,ab,kw  #3 #1 AND #2  #4 'diabetes':jt AND 'conference abstract':it AND '82nd scientific sessions of the american diabetes association, ada 2022':nc  #5 'diabetes':jt AND 'conference abstract':it AND '81st scientific sessions of the american diabetes association, ada 2021':nc  #6 'diabetes research and clinical practice':jt AND 'conference abstract':it AND 'idf virrual congress 2021':nc  #7 'diabetologia':jt AND 'conference abstract':it AND '58th annual meeting of the european association for the study of diabetes, easd 2022':nc  #8 #1 AND #4  #9 #1 AND #5  #10 #1 AND #6  #11 #1 AND #7  #12 #3 OR #8 OR #9 OR #10 OR #11 |
| Cochrane Library | #1 "clinical decision support system*" OR "cdss*" OR "decision support system*" OR "dss" OR "computer-assisted". ti ab kw  #2 "diabetes" OR "DM". ti ab kw  #3 #1 AND #2 |
| Web of Science | #1 (ti= ("clinical decision support system*" OR "cdss*" OR "decision support system*" OR "dss" OR "computer-assisted")) OR ab= ("clinical decision support system*" OR "cdss*" OR "decision support system*" OR "dss" OR "computer-assisted")  #2 (ti= ("diabetes" OR "DM")) OR ab= ("diabetes" OR "DM")  #3 #1 AND #2 |

**Appendix 1** (Continued)

| **Variables** | **Description** |
| --- | --- |
| CNKI | #1 主题="临床决策支持系统" OR 题目或关键词或摘要="临床决策支持系统"  #2 主题="糖尿病" OR 题目或关键词或摘要="糖尿病"  #3 #1 AND #2 |
| Wanfang | #1 主题:("临床决策支持系统") OR 题名或关键词:("临床决策支持系统") OR 摘要:("临床决策支持系统")  #2 主题:("糖尿病") OR 题名或关键词:("糖尿病") OR 摘要:("糖尿病")  #3 #1 AND #2 |
| VIP | #1 题目或关键词="临床决策支持系统" OR 摘要="临床决策支持系统"  #2 题目或关键词="糖尿病" OR 摘要="糖尿病"  #3 #1 AND # 2 |
| Google | #1 "clinical decision support system" OR "cdss" OR "decision support system" OR "dss" OR "computer-assisted"  #2 "diabetes" OR "DM"  #3 "International Diabetes Federation" OR "IDF" OR "American Diabetes Association" OR "ADA" OR "European Association for the Study of Diabetes" OR "EASD"  #4 #1 AND #2 AND #3 |
| Baidu | #1 "糖尿病"  #2 "临床决策支持系统"  #3 "中华医学会糖尿病学会" OR "CDS" OR "中华医学会内分泌学会" OR "CSE"  #4 #1 AND #2 AND #3 |
| Official conference websites | International Society for Pharmacoeconomics and Outcomes Research (ISPOR) official website  International Diabetes Federation (IDF) official website  European Association for the Study of Diabetes (EASD) official website |

ti: title; ab: abstract; kw: keyword; jt: journal name; it: publication type ; nc: conference name
