# Supplementary figures and images for: Applications of Clinical Decision Support Systems in Diabetes Care: Scoping Review
Source: J Med Internet Res. 2023 Dec 8;25:e51024. doi: 10.2196/51024 (PMC10746969; doi:10.2196/51024)

**Appendix 2.** Number of publications and subjects over time (N=85).


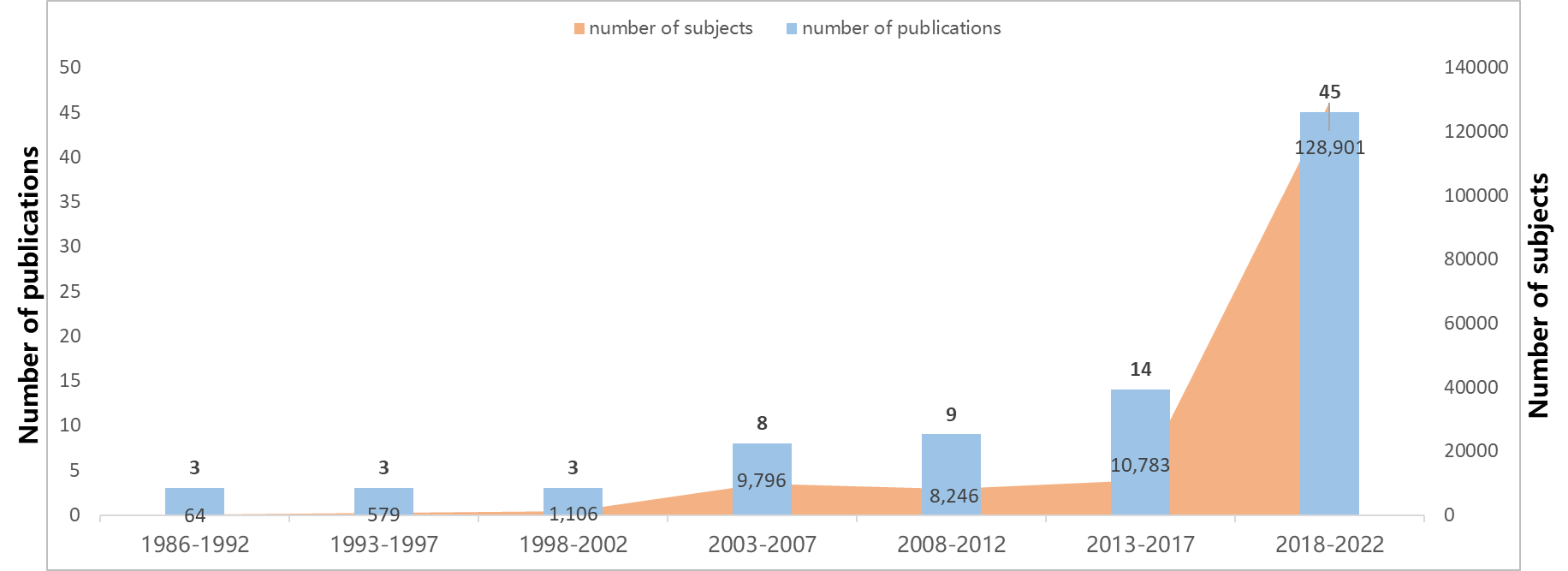

Supplement: Multimedia Appendix 3 [file jmir_v25i1e51024_app3.doc]
