# Supplementary material for: Applications of Clinical Decision Support Systems in Diabetes Care: Scoping Review
Source: J Med Internet Res. 2023 Dec 8;25:e51024. doi: 10.2196/51024 (PMC10746969; doi:10.2196/51024)
Supplement: Multimedia Appendix 4 [file jmir_v25i1e51024_app4.doc]

**Appendix 3. Summary of users’ adherence.**

| **Author** | **Type of user adherence** | **User** | **Results** |
| --- | --- | --- | --- |
| Spat et al. 2017^67^ | Suggestions for insulin dose adjustment | physicians | The physicians' adherence to the basal, bolus, and total daily insulin dose suggestions was 95.7%, 96.2%, and 98.7%, respectively. |
| Hochfellner et al. 2021^90^ | Suggestions for insulin dose adjustment | physicians and nurses | The users' adherence to the basal insulin dose suggestions was high on both days with fasting periods (95.3%) and days with regular food intake (96.4%), and the difference was not significant (p=0.642). ^a^ The users' adherence to the bolus insulin dose suggestions was significantly higher on days with regular food intake compared to days with fasting periods. (95.7% vs 91.6%, p<0.001) |
| Aberer et al. 2019^73^ | Suggestions for insulin dose adjustment | physicians and nurses | The physicians' adherence to the total daily insulin dose suggestions was 97.3%; the nurses' adherence to the basal and bolus insulin dose suggestions were 99.1% and 95.5%, respectively. |
| Lichtenegger et al. 2021^94^ | Suggestions for insulin dose adjustment | physicians and nurses | The physicians' adherence to the total daily insulin dose suggestions was 91.6%; the nurses' adherence to the basal and bolus insulin dose suggestions were 97.2% and 94.1%, respectively. |
| Neubauer et al. 2015^57^ | Suggestions for insulin dose adjustment | physicians and nurses | The physicians' adherence to the total daily insulin dose suggestions was 97.5%. The nurses' adherence to the basal and bolus insulin dose suggestions was 96.7% and 96.5%, respectively. The nurses' adherence to blood glucose measurements, bolus insulin injections, and basal insulin injections suggestions was 96.4%, 93.1%, and 98.6%, respectively. |
| El Fathi et al. 2020^80^ | Suggestions for insulin dose adjustment | physicians | The physicians' adherence to the insulin dose suggestions was 92% |
| Mader et al. 2019^77^ | Suggestions for insulin dose adjustment | physicians and nurses | The users' adherence to the insulin dose suggestions was 93% |

**Appendix 3** (Continued)

| **Author** | **Type of user adherence** | **User** | **Results** |
| --- | --- | --- | --- |
| Hannon et al. 2017^66^ | Follow-up/examination appointment | physicians | CDSS significantly improved the adherence to follow-up in children and adolescents at high risk for T2D (29.4% for CDSS group vs 18.9% for control group; adjusted odds ratio, 1.8; 95%CI, 1.5–2.2) |
| Holbrook et al. 2009^45^ | Follow-up/examination appointment | physicians and patients | 33% (11/33) physicians felt that patients' adherence with appointments had improved |
| Meigs et al. 2003^38^ | Follow-up/examination appointment | physicians and nurses | CDSS significantly improve the patients' adherence to HbA1c test (change in mean number of HbA1c tests obtained per year; +0.3 for CDSS group vs -0.04 for control group; P=0.008), LDL cholesterol test (change in mean number of LDL cholesterol tests obtained per year; +0.2 for CDSS group vs +0.01 control group; P=0.02), and foot examination (percentage change in at least one foot examination in the last 12 months; +9.8% for CDSS group vs -0.7% for control group; P=0.003). Change was defined as an increase or decrease in levels or proportions comparing the intervention year to the preintervention year. |
| Lobach et al, 1997^33^ | Diabetes care guideline | physicians, nurses, and medical assistants | CDSS significantly improve the users' adherence to care guidelines for diabetes mellitus (32.0% for CDSS group vs 15.6% for control group; P=0.01) |
| Nilasena et al, 1995^31^ | Diabetes care guideline | physicians | Compared with the control group, physicians in CDSS group reported greater change from baseline of adherence to care guidelines for diabetes mellitus (+16.90% for CDSS group vs +16.40% for control group; no significant difference). |
| Murphy et al. 2020^83^ | Suggestions for drug usage | physicians and nurses | The physicians' adherence to antihypertensive and antihyperglycemic medication suggestions was 67% and 48%, respectively. |

**Appendix 3** (Continued)

| **Author** | **Type of user adherence** | **User** | **Results** |
| --- | --- | --- | --- |
| Prabhakaran et al, 2019^78^ | Suggestions for drug usage | physicians and nurses | Compared with the control group, patients in CDSS group reported greater adherence to antihypertensive (81.1% for CDSS group vs 57.9% for control group) and antihyperglycemic (82.4% for CDSS group vs 68.9% for control group) medication. However, there was no significant difference in adherence between the two groups. |
| Ware et al, 2022^108^ | Suggestions for lifestyle | patients | The patients' adherence to the self-care instructions (including reminders to record blood glucose and suggestions to diet) was 73.1%. |
| Singh et al, 2018^72^ | Suggestions for drug usage & lifestyle | physicians and care coordinators | Physicians' acceptance rates of CDSS recommendations related to glycemic, BP and LDL cholesterol control were 54.5%, 79.2% and 71.1%, respectively. CDSS recommendations included reinforce lifestyle counselling, continue with existing regimen, up-titration of medication, and addition of insulin. |

^a^ This study aimed to assess the impact of the use of CDSSs during fasting periods in hospitalized patients with type 2 diabetes. During the fasting periods, patients missed one or more meals. During the days with regular food intake, patients returned to the regular diet.
